# Supplementary material for: Skin coloration is a culturally-specific cue for attractiveness, healthiness, and youthfulness in observers of Chinese and western European descent
Source: PLoS One. 2021 Oct 28;16(10):e0259276. doi: 10.1371/journal.pone.0259276 (PMC8553160; doi:10.1371/journal.pone.0259276)
Supplement: Attachment — Submitted filename: Response to Reviewers.docx [file pone.0259276.s002.docx]

We are really grateful to both reviewers for the valuable feedback. We have revised our manuscript according to the comments and suggestions, and we would like to respond to the questions one by one in below.

- **Response to reviewer #2:**

**1. The literature can be further expanded, both in breadth and also depth, to better established the scene – and justify the proposed hypothesis. The authors should consider discussing the potential mechanism involved, in brief. Also, worth discussing the reasons for potential cultural difference – apart from reporting the mixed results.**

Thanks for the suggestions. We tried to add some discussion in the introduction part. Also see below.

“Some of the associations between skin colour and perceptual ratings are clearly grounded in physical skin changes, e.g. the negative association between Lightness (L*) and both physical and perceived age is linked to sun exposure over time (Mayes et al.). Other associations between skin coloration and preference ratings might reflect the aesthetic differences between western and eastern culture, which are likely to result from the development of multiple social and cultural factors over a long period of time. We therefore hypothesize that associations between skin coloration and preferences will depend on both, the ethnicity of the observer and the ethnicity of the viewed facial image.

The aim of our study was to establish (1) the strength of the association between skin coloration (L*,a*,b*) and the perceptual attributes (attractiveness, healthiness, youthfulness), and (2) whether the utilization of these skin coloration cues (L*, a*, b*) is modulated by the ethnicity of the observer, the viewed image, or both. We used a large set (n=80) of calibrated, high-resolution images of real human faces (Chinese and western Europeans) comprising a range of ethnicity-specific skin colours (lightness L*, redness a*, yellowness b*), without digitally manipulating skin colour or texture. Two groups of observers (Chinese and western Europeans) rated both sets of facial images along three dimensions: perceived attractiveness, healthiness, and youthfulness. ”

**2. It would be better if the authors can introduce the term LAB-C, in brief, before discussing the individual component using only the label/acronym.**

Thanks for the suggestion. Explanation of L*, a*, b*, C* has been added to the stimulus description, also see below.

“CIELAB colour space is a device-independent standard colour appearance space where skin colour is described by three dimensions: Lightness L*, Redness a* and Yellowness b* [28]. Additionally, chroma is used to roughly represent the saturation of colours. Skin colours of both western Europeans and Chinese images were plotted in the a*-b* chromaticity diagram as well as the Chroma-Lightness diagram.”

**3. The presentation (display) of the stimulus should be better described for clarity. What software did the researcher use? And how was the actual protocol like? This info seems to be immersed with the image preparation part.**

Thanks for asking the questions. To make it more clear to readers, the following information has been supplemented to the section of Photography and Image Processing.

“Subsequently, each facial image was digitally edited to remove the hair, ears, and any visible clothing and the image was then scaled to be in the centre of the screen. Finally, a mid-grey background (L*, a*, b* = 50, 0, 0) was set to display all the images. All the images were processed in MATLAB. ”

**4. Please refer to line 132 – Were the participants being asked to attend only to the skin colour part of the image and not the overall face? If so, justification may be needed for this unconventional approach considering that there is a substantial amount of evidence showing that face is being processed holistically. If that is not the case, the writer may need to rephrase the specific statement.**

Thanks for the suggestion. It would be good to give a clearer statement for the experiment instructions. Specific instructions have been added to the ‘experimental procedure’ part of the revised paper: “The following question was asked after the observation of each image, “based on the skin color, what attractiveness score (or healthiness score or the estimated age, depend on different sessions) you would give for the last image?”’ In our experiment, observers viewed each facial image and then rated the skin color of each image with respect to three perceived attributes: attractiveness, healthiness, and visual age. What we want to investigate in this study is the the effect of skin colour on perceptual ratings. We try to focus on the role of skin colour and, therefore, the observers were asked to make judgements based on skin color rather than other factors such as facial geometrical characteristics. Even so, such influence cannot be totally ignored in all cases related to subjective ratings, which has been discussed in the discussion session.

**5. It would be great if the writer can provide some simple justification about the sample size used – to ensure that it has enough power.**

Thanks for the suggestion and justification has been added. “The sample size for this study was determined using G-power 3.1 software package (Faul, Erdfelder, Buchner, & Lang, 2009), with a P-value of 0.05, power of 0.80, and medium effect size of 0.5. A total sample needed for this study was calculated to be 34 and 44 observers were finally enrolled.” We also overviewed studies included in the literature review part and the sample sizes are basically on the same order of magnitude.

**6. Refer to line 112 - It may be better if the author can explicitly report the mean values of LAB for the Asian and Caucasian faces used – instead of a generic description about the difference between the two sets of stimuli. Even the actual mean difference will be better.**

This is a good advice and we have added the mean values into the ‘Stimulus Description’ section to make things clearer. There are systematic mean differences in lightness and chromaticity between the two ethnic groups. Caucasian faces: L*, mean=59.0, SD=1.9; a*, mean=8.3, SD=1.4; b*, mean=14.1, SD=1.9; Chinese faces: L*, mean=55.0, SD=2.1; a*, mean=8.9, SD=1.2; b*, mean=16.9, SD=1.4. The specific colour, of each of the 80 images can be found in Fig 2.

**7. Please refer to line 206-210 – The Result can be better phrased for clarity. Phrases such as “highly significant” or “highest negative correlation” may not be the conventional way of reporting the result – or at least not specific enough and the point of comparison is not clear. Please consider rephrasing this section for clarity.**

Thanks for the reminder and the corresponding paragraph has been revised. Phrases such as “highly significant” or “highest negative correlation” have been avoided.

**8. Please refer to line 213 – I am not certain what the number 18 after the p values stand for.**

Since there are multiple comparisons (18 separate correlations were performed), the alpha level was adjusted using the Bonferroni correction method to α= 0.05/k, where α is the adapted significance level and k is the number of tests, to guard against type one errors associated with multiple testing. All significant correlations were still significant at Bonferroni adjusted a = (0.05/18).

**9. Please refer to line 218-220 – A stat may be needed to support the inference made about the higher agreement of own ethnic faces rating than of the other ethnic’s.**

Thanks for the comment. It may not be appropriate to draw the conclusion that higher consistency was statistically achieved within own-ethnicity. Corresponding statement has been revised that, both set of observers show high levels of agreement on the subjective ratings in their own-ethnicity faces and other- ethnicity faces.

**10. Line 221-223, please explain the number (22)(44) in the bracket?**

The number in the bracket represents for sample size, as mentioned in the table caption. There are 22 Caucasian observers and 22 Chinese observers, and 44 observers in total.

**11. Line 228, maybe reconsider if the term “deployed” is best to be used here. This is merely a suggestion.**

The term has been changed to ‘utilized’. But we are not sure about the reason behind the concern. Maybe it’s better if reviewer could give any explanation why or suggest the more appropriate term to use.

**12. Table 3 is incomplete and hard to be seen clearly. I would assume the authors will also need to report those stats with a not-significant result, at least in the table.**

Thanks for the comments and we have revised Table 3 (table 4 in revised paper) to show all the results, with significant results in bold. Hope it will give a clearer picture of the results.

**13. Line 240-242, not sure if the conclusion stated here is accurate – I would refrain from implying that one (aspect) is higher than another if the result is indicating otherwise.**

We have now clarified the statement on line 240-242 as follows:

“Ratings of attractiveness and healthiness are driven by the same facial cues (Table 4), which is also supported by the high correlations between attractiveness and healthiness ratings (Table 5), consistent with Han et al. (2018). However, these cues are utilized differently by Chinese and western European viewers. Chinese observers use skin lightness as a robust cue for judging both attractiveness and healthiness, but only when judging Chinese faces (Table 4). For Chinese viewers, redness (a*) is negatively associated with both attractiveness and healthiness but reaches significance (p<0.05) only for attractiveness (Table 4). In contrast, western European viewers associate an increase in yellowness (b*) with increased attractiveness and healthiness, but only when viewing images of Chinese faces (Table 4). ”

**14. Line 252-253 – the author reported that - “Experiments linking high-carotenoid diet to objective skin colour changes have produced inconclusive results.” However, (all) the evidence listed subsequently in the adjacent sentences seems to be indicating that the supplementation consistently induces an increment in skin yellowness. The inconclusive part, I assume should be more on redness (and the subjective rating of health)?**

Thank you for spotting this. The contentious issue is indeed the relationship between a high-carotenoid diet and a* rather than b*. This has now been clarified:

“Experiments linking high-carotenoid diet to objective skin colour changes have produced inconclusive results for redness (a*).”

**15. Line 260-263 – please consider rephrase this section, for better clarity.**

We have now rephrased this section as follows:

“Highly elevated skin yellowness levels are likely to reflect underlying health issues such as jaundice [39]. Therefore, one would predict that only those small changes in yellowness that reflect objectively measurable health benefits, to be positively associated with perceived health. This non-linear relationship between physical skin coloration changes and perceptual preferences supports the argument that associations between physical skin changes and perceptual attributes such as healthiness and attractiveness need to be investigated within a physically plausible parameter range. ”

**16. I would avoid using the comparative terms (highest, higher etc) especially when there is no obvious reason for doing so (hypothesis wise, theoretically wise). Instead of saying “highest correlation”, a better way to go may be to merely indicate the valence (positive) and the strength (weak, medium, strong) – see, for example, line 265**

Thanks for the reminder and the inappropriate terms has been revised.

**17. Discussion can be expanded and better organized – This section seems to be mostly descriptive (the who and what – e.g., who found what). I would expect some explanation about the potential mechanism here (the why and how) – at least in brief. For example, why is luminance being associated with higher perceived health (and/or physical health)? Why redness is being related to the increment in perceived age?**

We have now speculated on the relationship between lightness and youthfulness. In our opinion, the evidence for strong associations between redness and health/attractiveness is weak.

“In summary, the most robust positive associations were found between facial skin lightness (L*) and attractiveness, healthiness, and youthfulness, but only when Chinese observers judge facial images of their own ethnicity. These associations between ratings and skin lightness are grounded in known physical changes: a decrease of one L* unit is equivalent to a 10-year increase in age in female Chinese skin [40]. In contrast to previous studies, we find no evidence that facial redness is positively associated with perceived attractiveness or health. A possible explanation for this discrepancy with previous results is that we used naturally occurring skin coloration variations, instead of manipulating the facial images. We speculate that previous experiments may have overestimated these associations by using skin color manipulations well beyond the gamut found in non-manipulated images as well as changing the coloration of the entire face instead of specific areas.”

**18. Please refer to line 300 – the authors wrote ‘also failed to find any association between facial redness and objective health measures”. This may be implying that the authors were also measuring the objective health measures – which I assume is not the case.**

Thanks for correction. We didn’t investigate any physical health measures. We have corrected the manuscript:

“Our results are, however, consistent with recent studies using a large image data base of real female faces that did not find any association between facial redness and objective health measures, neither any positive association between redness and attractiveness [9].”

**19. Line 301, would it be better to explicitly cite the last name here – for clarity and ease of reading. This is merely a suggestion.**

Thanks for the suggestion. Names have been added in text: “…in contrast to previous reports with western European (Stephen et al., 2009a; Stephen et al., 2009b; Pazda et al., 2016; Stephen et al., 2012) [2,3,13,18] and Chinese observers (Han et al., 2018; Tan et al., 2019) [20,21]….”

**20. Line 301-302 may not be entirely accurate, as redness is not, as a whole, correlated with attractiveness.**

This has now been rephrased:

“Our results are, however, consistent with recent studies using a large image data base of real female faces that did not find any association between facial redness and objective health measures, neither any positive association between redness and attractiveness [9].”

**21. Please check the in-text citation on Line 331.**

Sorry for the mistake. The in-text citation has been corrected.

**22. Line 355-356, this argument needs to be rephrased – as it is unclear what do you mean by “manipulated facial images showed strong association”. You have mentioned the stimuli used here (facial images) but not the IVs and DVs.**

“Broadly speaking, rating experiments using manipulated facial images showed strong associations [2,3,12,19,20,49] between skin coloration and subjective ratings of health and attractiveness. ”

**23. Please check your reference list – a few items do not seem to be with complete info (e.g, item 2, 8, 16, 21, 22, 23, 28, 32, 44).**

Sorry for the incomplete info. References have been checked.

- **Response to reviewer #1:**

**General comments
- I would ask the authors to make their data and analysis script(s) available for the reviewing process; I could not find these at the provided link. I have several questions regarding the analyses (see below), some of which I might have been able to answer myself with access to the data**

Please find the data in file “Data_Facial Attractiveness.xlsx”.

Also added the data information at the end of the paper: The data associated with this research are available at https://pcwww.liv.ac.uk/~sophiew/skin.htm

**- Han et al. (2018) found effects of both stimulus and observer sex. I assume the authors did not test for such effects due to sample constraints, but this should be acknowledged somewhere**

Thanks for the comments. Justification has been added to the ‘Experimental Procedure’ part. It is true that sex effect was not tested in this study. The observers were not either told about the image sex or asked to judge the image sex when making judgements during the experiments. Since each facial image was edited to remove the hair, ears, and any visible clothing, the sex of the face was not that obvious or highlighted in this study.

**Materials and Methods
Stimuli:**
**- How were images selected from the LLSD? Demographics of stimulus set should be reported (could age of stimulus faces have had an effect on results?)**

Thanks for the question. There are over sixty facial images for each of the Chinese and Caucasian groups in LLSD. Facial images with age below 20 or above 40 were not selected, so all the faces are from people of the same age range between 20-40. Both males and females were included in the experimental materials. Totally, they are 40 Chinese facial images and 40 Caucasian facial images. The relatively large number of images is used to cover a wide variation of facial shape and facial features. The information has been added in the image preparation part.

**- What software was used to mask non-face cues, how were images scaled/centered (i.e. algorithmically or manually), and how were CIELAB coordinates calculated?**

Thanks for the questions. The relevant information has been added into the text. MTALAB was used to do the image processing and calculation. Each facial image was digitally edited to remove the hair, ears, and any visible clothing manually and the image was then scaled to be in the center of the screen. As shown in the below left image (from Wang et al., 2018), all the images were captured in the light booth and the distance from the participant to the camera was fixed, so the facial areas are similar in size. Finally, a mid-grey background (L*, a*, b* = 50, 0, 0) was set to display all the images. The mean color specification, in terms of CIELAB coordinates, of 80 test facial images (40 Chinese and 40 western Europeans) were calculated as the overall mean of each pixel in the facial area, excluding the mouth, nose, eyes, and eyebrows. These excluded areas were manually removed from each facial image as the below right image shows.

**Observers:
- How were observers recruited?**

The participants need to be over 18 years old with normal colour vision. This requirement and the Information Sheet of this study were included in the recruitment email. Email was used to recruit the participants from the University of Leeds and the researchers also posted the Information Sheet on the information board at several schools in the university to allow volunteers to initiate contact about the study. A colour-blindness test, by using the Ishihara Test, was used as a screening test prior to the experiment. For those who would like to have a check, an online Ishihara colour vision test link was also included in the recruitment email. For the one could not access the online test, the participants were welcome to come to the lab to do the double check.

**- It is stated Chinese observers had spent about 1-3 years in the UK – mean and SD would be more informative here**

We are sorry that we only asked for an approximate time that observers spent in the UK, but we don’t have the specific time span, thus we cannot provide the mean and sd info here.

**Procedure:
- Was the order of sessions randomized?**

No. The order of the three separate sessions was not randomised. In each session, the observers were told to rate the images with respect to one specific attribute. The order of 80 facial images presented in each session was all randomised.

**Statistical analysis:
- It is stated that “all interactions between the predictors were included”—this could be a bit clearer. Was a full factorial design specified? Only two-way interactions are reported in Table 2, while Table 3 and the text suggests three-way interactions were analysed (see also comments below)**

Thanks for the suggestions. We would like to make it clearer that for each DV, the **simple model** was set up with lightness (L*), redness (a*) and yellowness (b*) as continuous predictors and observer ethnicity and image ethnicity as categorical predictors but without interaction term; while the **full model** allowing for all interactions between color (L*, a*, b*) and ethnicity (observer ethnicity, image ethnicity). Code to implement linear mixed effects models in R show as below:

#model1: Attractiveness (simple model)

model1 <- lmer(Attractiveness~1+L+a+b+Im_Ethnicity+Ob_Ethnicity+(1|ImID)+(1|ObID),REML=F,data = rawdata)

#model1.1: Attractiveness (full model)

model1.1 <- lmer(Attractiveness~1+L+a+b+L*Im_Ethnicity*Ob_Ethnicity+a*Im_Ethnicity*Ob_Ethnicity+b*Im_Ethnicity*Ob_Ethnicity+(1|ImID)+(1|ObID),REML=F,data = rawdata)

#model2: Healthiness (simple model)

model2 <- lmer(Healthiness~1+L+a+b+Im_Ethnicity+Ob_Ethnicity+(1|ImID)+(1|ObID),REML=F,data = rawdata)

#model2.1: Healthiness (full model)

model2.1 <- lmer(Healthiness~1+L+a+b+L*Im_Ethnicity*Ob_Ethnicity+a*Im_Ethnicity*Ob_Ethnicity+b*Im_Ethnicity*Ob_Ethnicity+(1|ImID)+(1|ObID),REML=F,data = rawdata)

#model3: Age (simple model)

model3 <- lmer(Age~1+L+a+b+Im_Ethnicity+Ob_Ethnicity+(1|ImID)+(1|ObID),REML=F,data = rawdata)

#model2.1: Age (full model)

model3.1 <- lmer(Age~1+L+a+b+L*Im_Ethnicity*Ob_Ethnicity+a*Im_Ethnicity*Ob_Ethnicity+b*Im_Ethnicity*Ob_Ethnicity+(1|ImID)+(1|ObID),REML=F,data = rawdata)

Both two-way and three-way interactions were included in the full model. Tests of fixed effect of three-way interactions have been added in Table 2 (table 3 in the revised paper).

**Results
- It is not clear exactly which models were tested and how they were specified. Table 2 reports main effects and two-way interactions, while Table 3 is split by observer ethnicity. This only make sense if the respective three-way interactions were included in the main model and found to be significant, in which case the corresponding stats need to be reported. Moreover, Table 2 only presents F- and p-values, while Table 3 reports significant estimates. All of this makes it hard to evaluate results and the subsequent discussion of effects. Findings would also be easier to parse if they were presented separately for the three DVs. This extends to Tables 2 and 3: estimates (including 95% CIs) and corresponding stats should be presented together, and tables instead split by DV**

Thanks for the comments. The models are specified as above. Table 2 (table 3 in the revised paper) has been modified. Both two-way and three-way interactions between colour and ethnicity(observer or image) were tested. Table 2 (table 3 in the revised paper) gives the overall test of all the explanatory variables without focusing on individual levels. Both two-way and three-way interactions on the bottom half of the table are interactions between colour and ethnicity, thus the significant effects show that skin color is a culturally-specific cue for the three DVs (two-way interactions: skin colour is rated differently by different observers or when different images were viewed; three-way interactions: skin colour is rated differently by different observers and when different images were viewed). Since there are two categorical variables with interaction (2 ob x 2 im), we will need to look into the individual levels in Table 3 (table 4 in the revised paper), which are the parameter estimates for the fixed effects of each subgroup in the linear mixed-effect models. Follow the other reviewer’s suggestion, we have revised Table 3 (table 4 in the revised paper) to show all the results, with significant results in bold. Thus, it may be difficult to put the two tables together. Hope it will give a clearer picture of the results.

**- It would be helpful to include graphs of significant interactions of interest (either in the main manuscript or the supplemental material)**

Thanks for the suggestion. Appendix S1 (labelled based on the order of occurrence)has been added as the graphs to show the significant interactions below,

Significant interactions：

- Attractiveness: L*:Im, L*:Ob, a*:Ob, b*:Ob, a*:Im:Ob
- Healthiness: L*:Im, a*:Ob, b*:Ob
- Age: L*:Im, a*:Im, L*:Im:Ob

Appendix S1 Fig.1 The regression lines to show significant interactions in the linear mixed-effect model.

**- When describing results in-text, effect sizes should be reported in addition to p-values**

Table 3 now contains the F-value. Is that sufficient?

**- Results should maybe start with info on inter-rater agreement (since low inter-rater agreement would render any further analyses meaningless). In that section it is also stated observers showed higher agreement within their own ethnicity— as the numbers are only marginally different, I am not sure this warrants extra mention?**

Thanks for the suggestion. The ‘Consistency within and between ethnicities’ part has been moved to the beginning of the results section. It is true that the numbers are only marginally different. It may not be appropriate to draw the conclusion that higher consistency was statistically achieved within own-ethnicity. Corresponding statement has been revised that, both set of observers show high levels of agreement on the subjective ratings in their own-ethnicity faces and other- ethnicity faces.

**- Presenting correlations between perceptual ratings separately for Chinese and Western observers and stimuli suggests that associations between these sub-groups differed—was this tested? It looks like age was more strongly associated with attractiveness and health in Chinese compared to Western participants, but I am not sure differences between Chinese and Western images would actually reach statistical significance?**

That’s an interesting point. We tested those associations using Pearson’s correlation coefficients and got different significant levels for Chinese and Western observers. Those P values can be compared, but we haven’t tested whether the difference reaches statistical significance. We are not sure how to test that. If the reviewer could give some advice on that, we would love to have a try.

**Discussion**

**- Like the results, I feel the discussion would benefit from some restructuring. Again, I found it a bit hard to understand what exactly was found and how these results sit within the existing literature**

We have tried to structure the discussion around the dependent variables: Perceived Healthiness, Attractiveness and Perceived Age. A section is devoted to ‘redness’ and perceived health/attractiveness since this has been the focus of many previous studies.

**- Attractiveness and b*. The positive effect of b* on attractiveness that was found for Chinese faces as judged by Western observers is interpreted in the context of a Western preference for tanned skin. This begs the question why no such preference was found for Western faces? In addition, there is some evidence that tan/melanin pigmentation is less preferred compared to carotenoid pigmentation. While there likely is some perceptual overlap between the two, the tan explanation does not appear entirely satisfactory. The authors also appear to doubt that high-carotenoid intake is linked to changes in skin pigmentation (line 252), but cite several studies that show such a link? Lastly, b* does not necessarily have to be linked to actual health in order to affect perceived health (or attractiveness); other mechanisms have been suggested (e.g., Han et al., 2018). I would be interested to hear the authors’ thoughts on this.**

This has now been clarified (see also R1, #14): “Experiments linking high-carotenoid diet to objective skin color changes have produced inconclusive results for redness (a*), but consistent results for b*.” It is correct that a strong relationship between b* and a physical health change is not a prerequisite for an association between b* and perceived health/attractiveness. However, our understanding is that the main argument in the literature is that **skin colour signals an underlying physical (**or mental) state. One would therefore predict that associations between skin coloration and perceptual attributes (perceived health/attractiveness) can only develop robustly if there is a **real and robust signal (information)** in skin coloration.

**- Effects of L*. In line with the current study, Han et al. (2018) found a stronger association of L* and attractiveness/perceived health for Chinese compared to Western observers. This also appears consistent with the current finding that L* was linked to perceived age in Chinese observers (especially since ratings of age seem to be quite strongly correlated with ratings of attractiveness and perceived health in Chinese observers). However, Han et al. reported an effect for both Chinese and Western faces, whereas the current study only appears to have found such an effect for Chinese stimulus images; I wonder whether this might have to do with different base levels of L* in unmanipulated images of Chinese and Western faces? Again, I would be interested to hear the authors’ thoughts on this.**

Thank you for the interesting comment. Chinese skin is on average slightly darker than western skin and the variability in L* is higher in western skin compared to Chinese skin (Xiao et al, 2016, SRT). It could be that some weber law behaviour is operating here. The other possibility is that the smaller variation in lightness in Chinese faces results in a more reliable cue for Chinese faces compared to western faces. A direct comparison between the Han et al. and our study is difficult due to different experimental and analysis methods, but the above argument is consistent with the idea that skin coloration cues are only informative, hence useful, if the variation is small. Ultimately, whether the lightness cue is a useful predictor for attractiveness/perceived health will therefore partly depend on the stimulus set used.

**- While I agree with the authors that colour cues are perceptually integrated with other facial cues (such as shape), I felt in its current form the section on “the role of other facial cues” was a bit unclear: what are the implications for the current study/findings?**

Fink’s study shows that an isolated facial skin patch (without the entire face) is a poor predictor for perceived age, health and attractiveness, compared to the information contained in the entire face; the R^2 between the ratings based on an isolated skin patch and entire face are 0.31, 0.14 and 0.06 respectively, clearly indicating the involvement of other variables. We have now added the following paragraph:

“Our current study was designed to estimate the strength of association between mean skin coloration (L*, a*, b*) and the perceptual ratings of perceived health, attractiveness, and perceived age; it does not allow us to estimate the contribution of skin coloration relative to the contribution of other cues, such as skin texture, localized coloration changes or other anatomical facial features. A useful follow-up study would be to include in the linear fixed-effect model spatial location and skin texture as independent variables, which would allow us to evaluate the relative contributions of mean skin coloration vs 2nd-order skin characteristics.”

**- Effects of a*. The discussion in this section switches in terminology to referring to “youthfulness” instead of age, which I thought was slightly confusing. It is reported that a* negatively affected attractiveness (lines 302 and 358); it should be clarified that this effect was only found for Chinese observers rating Western images. The authors provide some ideas as to why a* was not positively linked to attractiveness and health as in (some) previous studies; I was wondering whether these null- and negative findings on Western images in particular might also be linked to the association of a* and perceived age in the Western image set?**

Thank you for the insightful comments. We were initially also surprised by the lack of a positive association between a* and health/attractiveness. However, surveying the more recent literature using non-manipulated faces revealed a remarkable consistency with our findings. We believe that the discrepancy between these earlier and later studies is due to methodology, i.e. using manipulated vs intact facial images, as explained in the discussion. We also agree that the lack of robust positive associations between a* and attractiveness/healthiness is probably linked to the strong association between redness and perceived age, at least for WE images.

**Minor comments:
- Table 2 is labeled main effects but also presents interaction effects**

Sorry for the mistake. The table title has been corrected.

**- Table 3 should specify whether the reported estimates are standardized or not**

They are unstandardized estimates.

**- Tense sometimes switches between past and present in results**

Thanks for the comments. Tense has been checked.

**- While most references are** **indexed by numbers, a few are referred to in the FirstAuthor et al. format**

Thanks for the correction. References in-text have been checked and corrected to indexed format by numbers.

**- Supplemental info is labeled a bit confusingly; Figs. 1-3 are labeled as S1, and then there is an unnumbered Fig. that is labeled as S2. In addition, it would be good to add a bit more info to figure captions**

Sorry for the confusing. Si Fig 1 is added to show significant interactions in the linear mixed-effect models. Figs. 2-4 (original figs. 1-3) are labelled as S2, and Fig. 5 (original fig.) has been labelled as S3, if that makes it clearer. The figure captions are listed in the end of the manuscript, also see below.

# S1 Fig 1. The regression lines to show significant interactions in the linear mixed-effect model.

**S2 Fig 2.** The associations between perceived attractiveness and skin color. L* (top), a* (middle), b* (bottom); Western European observers (left column), Chinese observers (right column). The regression lines were drawn for the significant fixed effects in the linear mixed-effect model.

S2 Fig 3. The associations between perceived healthiness and skin color. L* (top), a* (middle), b* (bottom); Western European observers (left column), Chinese observers (right column). The regression lines were drawn for the significant fixed effects in the linear mixed-effect model.

S2 Fig 4. The associations between perceived age and skin color. L* (top), a* (middle), b* (bottom); Western European observers (left column), Chinese observers (right column). The regression lines were drawn for the significant fixed effects in the linear mixed-effect model.

S3 Fig 5. Associations between attractiveness, healthiness and age ratings for western European observers (left column) and Chinese observers (right column). The regression lines were drawn for the significant correlations at Bonferroni adjusted α (for details, see Table 4).
