# Supplementary material for: Skin coloration is a culturally-specific cue for attractiveness, healthiness, and youthfulness in observers of Chinese and western European descent
Source: PLoS One. 2021 Oct 28;16(10):e0259276. doi: 10.1371/journal.pone.0259276 (PMC8553160; doi:10.1371/journal.pone.0259276)
Supplement: Attachment — Submitted filename: Response to Reviewers.docx [file pone.0259276.s004.docx]

**Response to Reviewer #1:**

**• The authors have now shared their data file, which is great! I wonder whether they also plan to share their R analysis script? This would make it much easier to check analyses and suggest any potential improvements. I also note that I still could not find any data specific to the manuscript under the link they provide**

Response: Yes, we are happy to share our data as well as the R script. Both can be found at the website now.

**• The authors have now added some notes on sex of the images in the experimental procedure. I would still ask them to explicitly note this as a limitation of the current study; I’m not convinced that masking non-face cues actually also effectively masks any cues to sex/makes sex irrelevant in perception**

Response: Follow this suggestion, we now have added a separate paragraph in red in the end of the revised paper to point out this limitation of this study, also see below:

The effect of ethnic difference has been investigated in our experiments, using observers of both genders. It must be recognized however, that the gender of the observer is a possible additional variable in the observations. Previous studies found a strong effect of facial redness that impacts on perceived health and attractiveness for both male and female skin by skin color manipulations [56,57]. Whether there is a perceptual difference for other colour properties or facial colour appearance between gender and, if there is, how large the effect is in the realistic skin model compared to the cultural difference, requires further work.

**• The authors have explained in their response that all faces were between the ages of 20 and 40. Just to clarify, did they use all faces that fell within this age bracket, or were there any other criteria for inclusion/exclusion (so if someone wanted to replicate this study using the same stimulus set they’d have all the info they needed)? Would it maybe be possible to report mean age of the used faces?**

Response: Thanks for the careful consideration. When we captured the facial images, we didn’t ask participants the specific age but let them choose the age group they belong to. Originally, we collected facial images of 6 age groups (below 20, 20-29, 30-39, 40-49, 50-59, above 60), and we only used facial images from the age group of 20-40 for this study. Therefore, it may be difficult to calculate the accurate mean age of the used faces. For the criteria of inclusion/exclusion, as we described in the paper, only facial images with neutral facial expression were selected (any other images with a smiling face, mouth open, etc. were not used). We have also mentioned in the paper how we mask non-face cues and scale these images and that we performed a rigorous process of color characterization for both camera and display to truly present the color appearance of those facial images to observers, which are all essential points if someone want to replicate the facial stimulus.

**• It still wasn’t entirely clear to me whether masking and scaling were done manually or using some sort of algorithm. Either is fine, but in the interest of reproducibility, this could be explained in more detail**

Response: Thanks for the suggestion. The process of masking non-face cues and scaling into the centre of screen were done manually in MATLAB. The corresponding sentences were modified in red in the revised paper to explain things more clearly, also see below:

Subsequently, each facial image was edited to remove the hair, ears, and any visible clothing manually and the image was then scaled to be in the centre of the screen. Finally, a mid-grey background (L*, a*, b* = 50, 0, 0) was set to display all the images. All the images were processed in MATLAB. Fig 1 shows an example of a Chinese real facial image used in this study.

**• Analyses/reporting of results. The paragraph on Statistical Analysis (lines 167 ff) should include info on effect-coding (how were image- and observer-ethnicity coded). Table 3 should also include estimates and 95% CIs (I can provide code for that if the authors want). Minor point, but p-values <.001 should be reported as such (instead of p=.000), and leading 0s should be omitted.**

Response: Following the reviewer’s suggestions, we have added information of categorical variable coding into the paragraph on Statistical Analysis and this info can also be found in the R script we shared: Deviation coding was used to convert both image ethnicity and observer ethnicity into deviation-coded factors (code ‘-0.5’ for western European images/observers and code ‘0.5’ for Chinese images/observers) for testing the main effects of each model.

We also added the corresponding estimates, SE, 95% CIs in Table 3-5 (original Table 3 has been split by DV as suggested by reviewer #1). The 95% CIs was computed using the function confint() in lme4 package. We have also changed the way of reporting P=0.000 into p<0.001. Many thanks for these kind suggestions.

**• I recommend breaking up reporting of the three DVs into separate subsections to make them easier to digest. Similar to my comment in the previous round, Table 2 should include corresponding estimates, and analyses should not be split by image and observer ethnicity (as for Table 4, and interpretation of effects). For example, re the significant two-way interactions of L*/b* x observer ethnicity on attractiveness: There is a positive effect of l* for Chinese but not Western observers; there is a positive effect of b* that’s even stronger in Western faces; but the model does not indicate these effects differ significantly for Chinese and Western observers. The three-way interactions are easily enough explained by visualizing them—I’ve attached an example of the sort of graph I have in mind (left panel Western observers, right panel Chinese observers)**

Response: Thanks for the recommendations. We have followed the advice that breaking up reporting of the three DVs into separate tables (Table 3-5) and paragraphs and the tables have now included the corresponding estimates, 96%CIs, etc. The interpretation part has been modified accordingly in the revised manuscript in red.

Thanks reviewer #1 for providing the graph that visualize the interactions. In fact, we have provided the same graphs in S1 Fig 1 of Supporting Information. The regression lines in S1 Fig 1. Should be able to clearly show all the interactions between colour and ethnicity (observer/image) in the linear mixed-effect model.

Since Table 3-5 reveal the main effects and there are significant interactions between colour and ethnicity, to further understand these interactions and reveal the simple effects of L*, a* and b* within each set of image and observer, we computed parameter estimates for the fixed effects within each subgroup from the three linear mixed-effect models, as shown in Table 6. Although reviewer #1 recommended us to report the main effects in results split by DVs, as we did for the modified Table 3-5 in revised paper, we still think table 6 (original table 4) has some value in revealing the effect of skin colorations at each level of the ethnicity factors in this study and thus we hope to keep this table after table 3-5.

**• The authors have now included F-values in Table 3, but not really addressed my comment—I meant that, e.g., on line 209, “interaction between image ethnicity and lightness was significant (p=0.017)” should instead be “(estimate=0.28, 95%CI [0.06, 0.51], t78.74=2.45, p=.017)”**

Response: Sorry for the misunderstanding of this comment in the previous round. We have now included all these information including the estimates, 95%CIs, t-value, etc. in Table 3-5 following the examples given by reviewer #1. We believe the current version has addressed the comments from reviewer #1.

**Response to Reviewer #2:**

**1. Argument on Lines 73-76 should be supported with evidence.**

Response: Thanks for the comments. We have added relevant references [23-26] in text.

**2. For attractiveness, I reckon there is a significant effect of image ethnicity? Please check. See Lines 207-210 and Table 3.**

Response: Yes, there is a significant effect of image ethnicity(p=0.023). Thanks for the reminder and the corresponding statement has been modified in the revised paper.

**3. For estimated age, is there is a significant interaction between the ethnicity of the image and observer? If so, is there any reason why it was not being included in the writing?**

Response: Yes, there is a significant interaction between the ethnicity of the image and observer (p=0.048) in the age model and we have now added the corresponding statement in the revised paper as well. For the further analysis and discussion part, the current study addressed the effect of skin colours (L*,a*,b*) on perceptual attributes (attractiveness, healthiness, youthfulness) and discussed how these colour cues are used differently by different observers and when viewing different faces. Therefore, only interactions between colour cues and ethnicity(image/observer) were mainly discussed, not the interaction between ethnicity and image.

**4. Western European observers associate an increase in yellowness with healthiness – only when viewing facial images of Chinese. Can you please check again your writing in the Result: Lines 232-233 (or maybe Lines 232-235, as a whole).**

Response: Thanks for the careful check. We are sorry there is a missing part in this sentence. We have now double checked the results and corrected this sentence in the revised paper. Also see below: Western European observers associate an increase in yellowness with healthiness (p=0.021) when viewing facial images of Chinese.

**5. In Discussion, Line 276-277, the claim made here should only be relevant for the viewing of the European face. Please check.**
Response: Thanks for the careful review. Yes, the statement has been modified to restrict with the viewing of the European face. Also see: For Chinese viewers, redness (a*) is negatively associated with both attractiveness and healthiness but reaches significance (p<0.05) only for attractiveness when viewing European faces (Table 6).

**6. For the Discussion, the authors have not properly fixed the carotenoids-yellowness-redness linkage. The authors start their claim by saying that the observed change in skin redness with** **the consumption of carotenoids was not consistent (in the lit). They then reported only those studies that observed NO significant change in skin redness (with the consumption of carotenoids). Hence the evidence presented does not seems to be matching the core argument. Perhaps this paper can be used to illustrate the “inconsistent” part -** **<https://pubmed.ncbi.nlm.nih.gov/26186449/>**

Response: Thanks reviewer #2 for providing this useful study. we have added this reference ([46]) and the relevant statement has been fixed to revealing the inconsistent results of how the consumption of carotenoids influence the observed change in skin redness. Also see below:

Experiments linking high-carotenoid diet to objective skin color changes have reported the consistent associations between carotenoids intake and skin yellowness (b*), however, the results for the carotenoids-redness (a*) linkage are inconclusive. Fruit and vegetable (FV) intake has been associated with a significant increase in skin yellowness (about 2 b* units, much smaller in a* values) and perceived attractiveness, consistent with the putative carotenoid-linked health-signaling system for mate choice [12,44]. Pezdirc et al [45] reported an increase of 0.6 b* but no measurable increase in redness (a*), which was confirmed by Appleton et al. [8] in a randomized controlled trial where they documented a small but significant effect of FV intake on skin yellowness (about 1-2 b* units), but no change in skin redness and no effect on perceived health. On the contrary, Tan et al. reported significant increments in both skin yellowness and redness (p<0.001) after 4-week FV intervention [46].

**7. Also, since carotenoids colouration is related to both skin yellowness and skin redness – I would suggest the author start the sub-section by saying that – “even though the association between carotenoids consumption and skin yellowness has been consistently reported in previous studies, but such consistency cannot be applied to the linkage between carotenoids intake and skin redness (with their phrasing of course). It seems awkward to include a claim merely highlighting skin redness in between two chunks of discussion about skin yellowness. See Lines 277-301 but specifically also the claim made on Lines 287-288.**

Response: This is the comment for the same part as the last comment. We agree with the reviewer’s opinion, and we have adopted this suggestion to make the relevant paragraph more coherent. Also see the revised paragraph in above. We think now the evidence presented is matching the core argument we make here.
